# Advancing Nanotechnology: Targeting Biofilm-Forming Bacteria with Antimicrobial Peptides

**DOI:** 10.34133/bmef.0104

**Published:** 2025-03-04

**Authors:** Julia Valladares Campos, Janaína Teixeira Costa Pontes, Christian Shleider Carnero Canales, Cesar Augusto Roque-Borda, Fernando Rogério Pavan

**Affiliations:** ^1^ São Paulo State University (UNESP), Tuberculosis Research Laboratory, School of Pharmaceutical Sciences, Araraquara, Brazil.; ^2^ Facultad de Ciencias de la Universidad Nacional de Ingeniería, Lima, Peru.; ^3^ Vicerrectorado de Investigación, Universidad Católica de Santa María de Arequipa, Arequipa 04000, Peru.

## Abstract

Nanotechnology offers innovative solutions for addressing the challenges posed by biofilm-forming bacteria, which are highly resistant to conventional antimicrobial therapies. This review explores the integration of pharmaceutical nanotechnology with antimicrobial peptides (AMPs) to enhance the treatment of biofilm-related infections. The use of various nanoparticle systems—including inorganic/metallic, polymeric, lipid-based, and dendrimer nanostructures—provides promising avenues for improving drug delivery, targeting, and biofilm disruption. These nanocarriers facilitate the penetration of biofilms, down-regulate biofilm-associated genes, such as ALS1, ALS3, EFG1, and HWP1, and inhibit bacterial defense mechanisms through membrane disruption, reactive oxygen species generation, and intracellular targeting. Furthermore, nanoparticle formulations such as NZ2114-NPs demonstrate enhanced efficacy by reducing biofilm bacterial counts by several orders of magnitude. This review highlights the potential of combining nanotechnology with AMPs to create novel, targeted therapeutic approaches for combatting biofilm-related infections and overcoming the limitations of traditional antimicrobial treatments.

## Introduction

Biofilm-forming bacteria represent a substantial global health concern due to their inherent resistance to conventional antibiotic therapies [1]. Biofilms are structured communities of microbial cells encased in a self-produced extracellular polymeric matrix, allowing them to adhere to surfaces and evade the host immune system [2]. These microbial structures are responsible for up to 80% of chronic infections, contributing to a wide range of medical conditions, including respiratory infections, endocarditis, and device-related infections [3]. Biofilm-forming pathogens, such as *Pseudomonas aeruginosa* and methicillin-resistant *Staphylococcus aureus* (MRSA), are particularly challenging to treat due to their capacity to withstand high doses of antibiotics, rendering many standard treatments ineffective [4].

Nanotechnology has emerged as a powerful tool in modern medicine, offering innovative approaches for diagnosing, delivering therapeutics, and overcoming the barriers presented by bacterial biofilms [5]. By utilizing nanoscale materials with unique physicochemical properties, researchers can develop systems capable of targeting bacterial cells more effectively, enhancing the bioavailability of drugs, and improving treatment outcomes. In the pharmaceutical field, nanotechnology enables the creation of drug delivery systems that can penetrate biofilms, release antimicrobials in a controlled manner, and reduce the toxicity associated with traditional antibiotics [6].

Antimicrobial peptides (AMPs) are a promising class of bioactive molecules that can range from 2- to 100-amino acid residues in length, can be either charged or uncharged, and may be synthetic or produced naturally by cellular machinery [7]. They exhibit broad-spectrum activity against various pathogens, including bacteria, fungi, and viruses [8]. These peptides act by disrupting microbial membranes, inhibiting quorum sensing, and interfering with biofilm formation by inducing cell aggregate dispersion, reducing adhesion, attenuating extracellular polymer production, and negatively regulating transporter expression [9–12].

Their ability to target multiple bacterial pathways, unlike conventional antibiotics that typically focus on one specific target, significantly lowers the likelihood of bacteria developing resistance to these compounds [13–15]. Furthermore, AMPs exhibit potent activity against Gram-negative bacteria, play an essential role in the host’s innate immune defense system, and act rapidly to kill bacteria [16] while also resensitizing bacteria to conventional antibiotics [17]. When combined with nanotechnology-based delivery systems, AMPs can overcome the limitations of poor stability, rapid degradation, and low bioavailability that often hinder their clinical application [9,18].

This review focuses on the intersection of pharmaceutical nanotechnology and AMPs for targeting biofilm-forming bacteria. It explores various nanoparticle platforms—including metallic, polymeric, lipid-based, and dendrimeric nanocarriers—that enhance AMP stability, promote biofilm penetration, and improve antimicrobial efficacy. Additionally, we discuss the challenges of treating biofilm-associated infections, mechanisms of bacterial resistance, and the potential of nanotechnology to revolutionize current therapeutic strategies. By understanding the synergies between nanotechnology and AMPs, this review aims to provide insights into the development of more effective treatments for biofilm-related infections.

## Nanotechnology in Pharmaceuticals

Nanotechnology refers to the design, creation, and application of devices, systems, and materials that function at the nanometer scale, offering practical solutions in a variety of fields [19]. This technology enables the development of biological, physical, and chemical systems that operate at submicrometric levels, allowing interactions with biomolecules and advancing our understanding of biological processes [20]. Nanotechnology holds the potential for early detection of biomarkers, targeted drug delivery, and specific interactions with cells and tissues, proving valuable in the treatment of infectious diseases [21,22].

In modern medicine, the development of nanotechnology has paved the way for innovative applications. Nanotechnology-based devices have been utilized in the creation of medical robots, and others have been developed to modify and detect potential diseases within the human body, enabling early diagnosis. Additionally, nanomaterials are increasingly being employed in regenerative therapies, such as cell therapy, tissue regeneration, and gene sequencing, to improve outcomes in the treatment and repair of cells, tissues, and organs [23].

Pharmaceutical science is one of the fields that have greatly benefited from nanotechnology, particularly in the design of advanced drug delivery systems. These systems offer higher precision and safety by targeting specific pharmacological sites, thereby reducing toxicity and improving the economic viability of treatments [20]. Nanostructures have been tested in a variety of therapeutic applications, including inhalation therapy for tuberculosis, the development of vaccine implants for sustained drug release, and materials capable of crossing the blood–brain barrier to treat diseases such as Alzheimer’s [24].

Nanoparticle-based drug carriers typically range in size from 1 to 1,000 nm and can be fabricated in 2 main ways: either by incorporating the drug into the nanoparticle during formulation or by adsorbing the drug onto the nanoparticle surface after formulation through incubation with a concentrated drug solution [25,26]. Drug delivery nanoparticles are categorized into several types, including carbon nanotubes (CNTs), nanowires, nanoshells, dendrimers, liposomes, niosomes, and nanorobots [27]. By selecting appropriate matrix materials, it is possible to modulate drug release, enhance drug stability, and achieve targeted delivery, making nanotechnology a promising alternative for novel therapeutics [26].

Infections caused by biofilm-forming bacteria present a significant global health challenge due to the difficulty of achieving effective treatment. According to Gao et al*.* [28], conventional antibiotic therapy often proves inadequate for treating chronic lung infections caused by *P. aeruginosa* in cystic fibrosis patients, as these bacteria can form biofilms. Many clinically important bacteria are biofilm-formers and are associated with infectious diseases such as vaginitis, periodontitis, colitis, urethritis, conjunctivitis, otitis, and implant-associated infections, accounting for 80% of infectious diseases [29].

Biofilms serve as highly effective bacterial resistance mechanisms by forming dense, protective layers that can block antibiotic penetration, modulate enzyme production, and activate efflux pumps [30]. As a result, bacteria embedded in biofilms exhibit 10- to 1,000-fold higher resistance to antibiotics compared to planktonic bacteria [31]. The widespread prevalence of multidrug-resistant bacteria is closely linked to biofilm formation, and nanotechnology offers a promising approach to addressing biofilm-related infections by enabling nanoparticles to penetrate biofilms and target infected sites more effectively, enhancing the bioavailability of antibiotics while minimizing side effects [28]. This article explores recent advances in pharmaceutical nanotechnology for combating biofilm-forming bacteria, combined with a novel therapeutic approach: AMPs.

## Understanding Biofilm Formation

### Characteristics and properties of bacterial biofilms

**Fig. 1.**
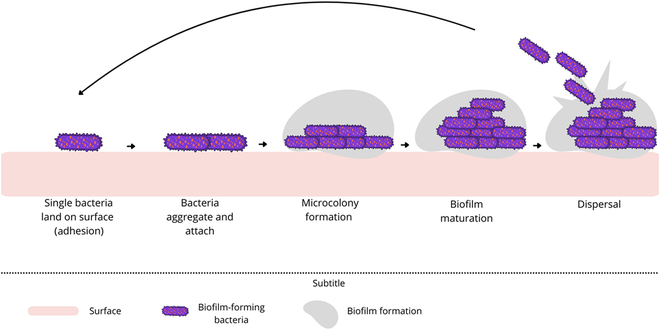
Stages of bacterial biofilm formation. The diagram illustrates the sequential phases of biofilm formation, starting with the adhesion of individual bacteria to a surface, followed by bacterial aggregation and attachment, microcolony formation, biofilm maturation, and, finally, bacterial dispersal to new surfaces.

Bacterial biofilms are complex, multilayered communities of cells attached to surfaces and encased in a matrix of extracellular polymeric substances (EPSs), which provides a protective environment for bacterial survival [32]. The EPS matrix plays a critical role in biofilm integrity, enabling bacteria to withstand hostile conditions, antibiotics, and physical stressors. Biofilms can form on a variety of surfaces, including organic and living matter such as tissues and cells [33]. Environmental factors like pH, temperature, and nutrient availability also significantly influence biofilm formation (Fig. 1) [34].

Biofilm-forming bacteria exhibit several distinctive traits:

• Quorum sensing, using signaling molecules to regulate biofilm development and communication between cells [35];
• Cyclic nucleotide second messengers, which modulate biofilm formation and dispersal by regulating bacterial motility, adhesion, and EPS production [35];
• Matrix-associated proteins, which enhance biofilm stability and structural integrity [35].

Despite their varied composition, biofilms share a common feature: They are highly hydrated, comprising approximately 97% water. Water channels within the biofilm structure play a vital role in nutrient transport and waste removal [34,35].

The process of biofilm formation can be divided into 5 stages: adhesion, irreversible adhesion, microcolony formation, biofilm maturation, and dispersal [36]. Initially, bacteria adhere loosely to a surface, usually in a fluid environment. As the biofilm matures, it becomes more resistant to environmental stress [35,36]. When resources are depleted, bacteria disperse to colonize new sites [35].

### Mechanisms of antibiotic resistance in biofilms and economic impact

Bacterial biofilms exhibit a unique resistance to antimicrobial agents, driven by a combination of environmental, structural, and physiological factors. Within biofilms, bacteria can survive high doses of antibiotics, but once removed from the biofilm structure, they often regain susceptibility, underscoring the biofilm-specific nature of their resistance [37]. Key mechanisms of antibiotic resistance in biofilms include the following: (a) Reduced antibiotic penetration: The EPS matrix acts as a physical barrier, blocking or slowing the diffusion of antibiotics, such as aminoglycosides, and allowing time for other resistance mechanisms to activate [38]. (b) Enzymatic modification of antibiotics: Enzymes within the biofilm matrix can degrade or inactivate antimicrobial agents [39]. (c) Presence of extracellular DNA (eDNA): eDNA within the EPS contributes to structural stability and provides binding sites for antimicrobial agents, reducing their effectiveness [39]. (d) Hypoxia: Oxygen depletion in the biofilm core reduces the efficacy of oxygen-dependent antibiotics, such as β-lactams [40]. (e) Slow bacterial growth and metabolic dormancy: These states reduce the activity of antibiotics targeting actively dividing cells [39]. (f) Activation of efflux pumps: Bacteria in biofilms up-regulate efflux pumps, expelling antibiotics before they can exert their effects [39]. (g) Quorum sensing: This cell-to-cell communication system regulates gene expression, including those involved in antibiotic resistance and biofilm maintenance [39]. The persistence of biofilm-related infections underscores the need for new antimicrobial strategies that can overcome these barriers [40].

**Fig. 2.**
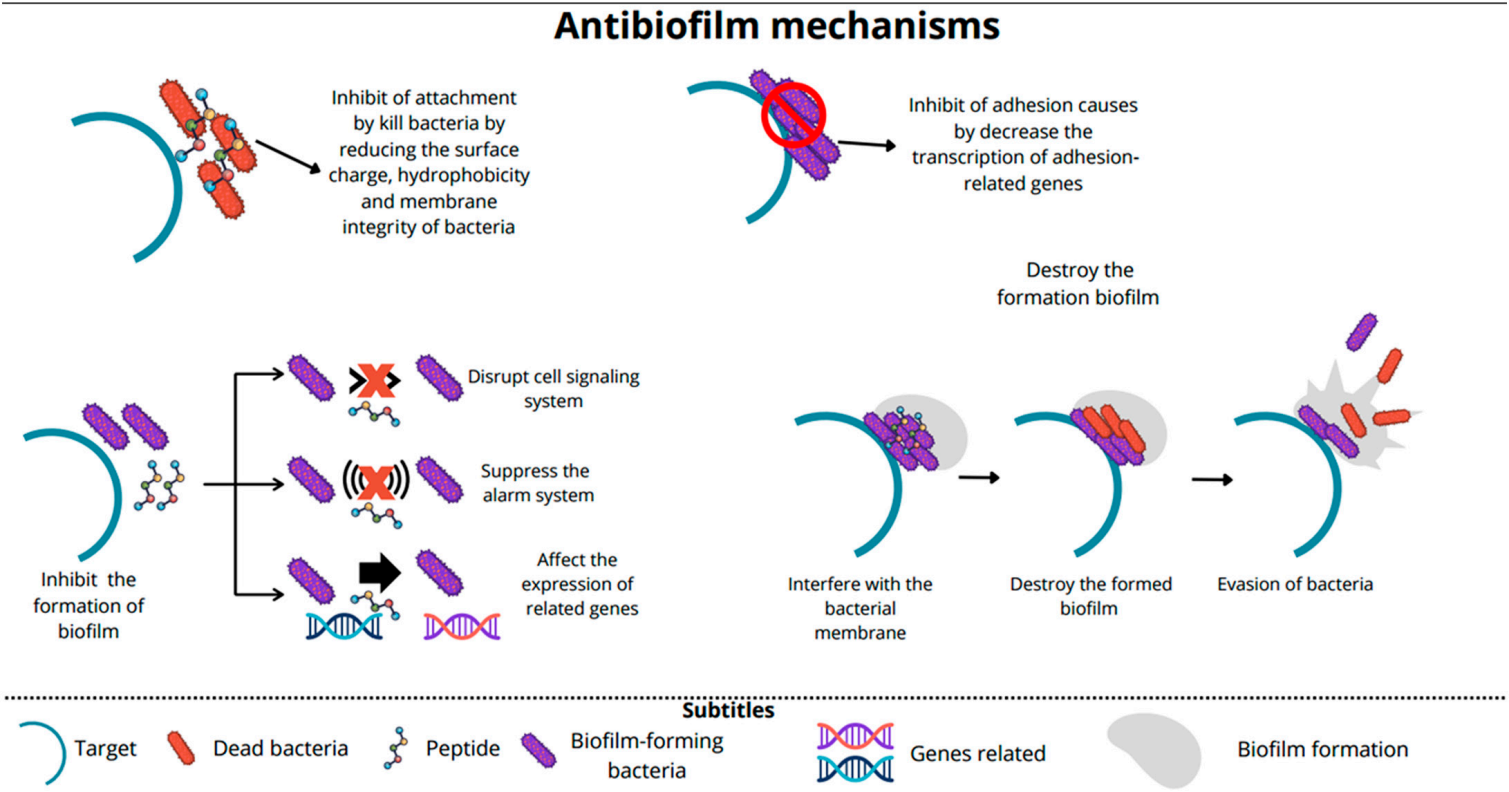
Mechanisms of action in biofilm inhibition and disruption by targeted interventions.

Biofilms pose significant public health challenges and economic consequences as studied before by Roque-Borda et al. [41]. An estimated 60 to 80% of bacterial infections are biofilm-related, occurring on medical devices such as catheters, pacemakers, and contact lenses, as well as in human tissues like the skin, eyes, and gastrointestinal tract [42,43]. Biofilm-associated infections are linked to chronic diseases (e.g., cystic fibrosis, chronic wounds, and osteomyelitis), device failures, and increased healthcare costs due to prolonged treatments and complications [44]. The economic burden extends beyond healthcare to industries like water distribution systems, where biofilms contribute to pipe blockages and contamination, further amplifying costs. Addressing biofilm-associated resistance is essential not only for public health but also for reducing economic losses across various sectors [44]. Several strategies have been proposed to inhibit or disrupt biofilm formation and enhance treatment efficacy (Fig. 2):

• Inhibition of attachment: By killing bacteria or altering their surface properties, such as charge, hydrophobicity, and membrane integrity, attachment to surfaces can be prevented and this reduces the initial formation of biofilms.
• Inhibition of adhesion: Reducing the transcription of genes associated with adhesion can prevent bacterial attachment to surfaces, thereby limiting biofilm development.
• Interference with cell signaling and gene expression: Disruption of cell signaling pathways impairs communication between biofilm-forming bacteria. Suppression of quorum sensing and other alarm systems further inhibits biofilm maturation, and altering the expression of biofilm-related genes can prevent their formation or stability.
• Membrane interference and biofilm disruption: Interventions may target and disrupt the bacterial membrane, leading to the destruction of biofilm structures and breaking down established biofilms results in bacterial evasion and dispersal, making bacteria more susceptible to treatments.

## Advances in AMPs

### Nature and mechanism of action of AMPs

AMPs are compounds composed predominantly of α-amino acids that exhibit antimicrobial activity or enhance the action of other antimicrobial agents when administered together [7]. AMPs can range from 2- to 100-amino acid residues in length, can be either charged or uncharged, and may be synthetic or produced naturally by cellular machinery [7]. These peptides are found in a wide array of sources, including bacteria, fungi, plants, insects, amphibians, fish, birds, mammals, and even the human body [10]. AMPs possess a broad spectrum of activity against bacterial infections, employing various mechanisms of action, including disrupting cell wall integrity, compromising cell membrane structure, modulating immune responses, targeting intracellular components, and inhibiting biofilm formation [45].

The bacterial cell wall serves as the first line of defense, and AMPs can compromise it through different mechanisms, such as binding to lipid II, which is essential for cell wall synthesis, inhibiting the biosynthesis of wall teichoic acids (WTAs), and promoting the release of autolysins, thereby weakening the bacterial defenses [46–48]. Membrane disruption, another key antimicrobial mechanism, can occur through 2 general pathways [45]. The first is due to the amphipathic nature of many AMPs, which allows them to insert themselves into bacterial membranes, forming pores or channels that disturb membrane integrity, leading to leakage of cellular contents and, ultimately, cell death [45]. The second, known as the “carpet model,” involves the peptide forming a layer over the membrane like a carpet, which disrupts the lipid bilayer, causing destabilization and microbial death [45]. AMPs are also effective in targeting biofilms through the mechanisms mentioned above, but additional strategies have been observed. These include quorum sensing inhibition, which disrupts bacterial communication, inducing the dispersal of cell aggregates, reducing adhesion, attenuating extracellular polymer production, and down-regulating transporter expression, all of which reduce biofilm formation [10–12]. Collectively, these mechanisms highlight the potential of AMPs in overcoming antimicrobial resistance and treating infections that no longer respond to conventional therapies [49].

### Advantages of AMPs over conventional antibiotics

In the context of rising antimicrobial resistance, AMPs stand out as valuable alternatives to traditional antibiotics. Their ability to target multiple bacterial pathways, unlike conventional antibiotics that typically focus on one specific target, significantly lowers the likelihood of bacteria developing resistance to these compounds [13–15]. Resistance mechanisms in bacteria often involve the acquisition of resistance genes, the activation of defensive strategies such as the production of enzymes that inactivate antibiotics, or the use of efflux pumps [15]. However, AMPs often bypass these mechanisms by targeting bacterial membranes, allowing them to evade traditional resistance strategies [13]. Furthermore, AMPs exhibit potent activity against Gram-negative bacteria, play an essential role in the host’s innate immune defense system, and act rapidly to kill bacteria [16].

AMPs have also demonstrated the capacity to resensitize bacteria to conventional antibiotics. According to studies by Martinez et al*.* [17], the use of AMPs in infections caused by *S. aureus* reduced resistance to vancomycin by enhancing the antibiotic’s permeability, thereby improving its likelihood of reaching its target, and facilitating successful treatment. Infections caused by microorganisms resistant to colistin and carbapenem have also been effectively treated using chromatin immunoprecipitation (ChIP), an AMP with a unique mechanism of action that does not rely on surface charge modifications [14]. This enables ChIP to bypass the primary cause of resistance to these antibiotics, making it an effective treatment option [14].

One of the most successful AMPs currently in clinical use is vancomycin, a synthetic tricyclic glycopeptide isolated from *Mycobacterium orientalis* [50]. Vancomycin has been in use for over 60 years and works by inhibiting bacterial cell wall synthesis, making it highly effective against Gram-positive bacteria [14,50]. It remains a first-line agent for treating infections caused by multidrug-resistant bacteria, and AMPs also have great potential in combination therapy, working synergistically with antibiotics to enhance treatment efficacy [51]. Combining different antimicrobials allows for lower dosages, reduces side effects, increases treatment selectivity, and targets multiple pathways simultaneously [51]. As reported by Knappe et al*.* [52], the efficacy of a drug in monotherapy can be significantly enhanced when it interacts synergistically with AMPs present in the body. Some applications were reported in Table 1.

**Table 1.** Antimicrobial peptides with biofilm inhibitory activities and their key functional highlights

AMP	Sequence	MIC	Highlights	Ref.
LL-37	LLGDFFRKSKEKIGKEFKRIVQRIKDFLRNLVPRTES	64 μg/ml	Reduces bacterial attachment, stimulates contraction movement, and affects 2 main quorum sensing systems	[92]
IDR -1018	VRLIVAVRIWRR	<1 μg/ml	Besides exhibiting immunomodulatory functions, IDR-1018 has the ability to eliminate biofilms of both Gram-positive and Gram-negative bacteria at concentrations much lower than its minimum inhibitory concentration against planktonic cells.	[93]
Pleurocidin	GWGSFFKKAAHVGKHVGKAALTHYL	16 μg/ml	Compared to the untreated biofilm, the biofilm mass decreased to 69%, 54%, and 30% when treated with 16, 64, and 256 μM pleurocidin, respectively.	[94]
BMAP-27	GRFKRFRKKFKKLFKKLSPVIPLLHL	2 μg/ml	At doses of ^1^/_8_ MIC, the peptide was able to inhibit up to 43.10% of the biofilm formed by *S. typhimurium*.	[95]
SMAP-29	RGLRRLGRKIAHGVKKYGPTVLRIIRIA	4–32 μg/ml	Among the 12 peptides tested in the study, only SMAP-29 and TP4 were able to inhibit the biofilm in the in vivo tests conducted with *Acinetobacter baumannii.*	[96]
D-LL-31	LLGDFFRKSKEKIGKEFKRIVQRIKDFLRNL	40 μg/ml	The peptide LL-31 was tested alongside its enantiomer D-LL-31, which exhibited better inhibitory activity against oral biofilms formed by *P. gingivalis.*	[97]
KSL-W	KKVVFWVKFK	25 μg/ml	The microemulsions loaded with KSL-W were used in the study and showed a significant reduction in the biofilm formation of *F. nucleatum*.	[98]
LI14	LKKLCRI	128 μg/ml	It exhibits potent antibacterial activity against all the tested resistant bacteria, in addition to excellent anti-biofilm activity and no toxicity recorded in vitro and in vivo.	[99]
S100A12	TKLEEHLEGIVNIFHQYSVRKGHFDTLSKGELKQLLTKELANTIKNIKDKAVIDEIFQGLDANQDEQVDFQEFISLVAIALKAAHYHTHKE	50 μg/ml	It inhibits the expression of genes involved in pyoverdine synthesis and biofilm formation.	[100]

### Challenges in the clinical use of AMPs

Despite their promising potential as new therapeutic agents, AMPs face several challenges in clinical applications. One of the main hurdles is their stability, as many AMPs have a relatively short half-life [14]. The average half-life required for Food and Drug Administration (FDA) approval is 50 h for large molecules and 37 h for small ones, and AMPs with shorter half-lives are not suitable for clinical use [53]. Moreover, AMPs are sensitive to environmental factors such as hydrolysis and photolysis, and they are prone to rapid degradation by proteases [14]. This limits their administration via intravenous routes, as this method also increases the risk of hepatic and renal clearance. AMPs also face poor mucosal penetration, making oral administration difficult [54].

In certain scenarios, AMPs that do not directly kill target bacteria but instead modulate the immune system can result in bacteriostatic effects, which may lead to higher concentrations of AMPs accumulating in the body [14]. This can result in side effects such as chronic inflammatory diseases [14]. Additionally, AMPs may cause hemolysis by disrupting the plasma membranes of erythrocytes [55]. The economic barrier is another factor to consider. As with any new drug development, the use of AMPs involves high costs for experimental trials and clinical testing, which often require extensive research time, making the investment less attractive from a commercial perspective [14].

### AMPs with antibiofilm activity and in vivo efficacy

The emergence of multidrug-resistant bacteria has driven interest in AMPs as promising candidates for developing natural antibiotics [56]. However, the therapeutic application of AMPs faces several significant challenges. One key challenge is the difficulty of replicating the organism’s complex environment in a clinical setting. Bacterial biofilms exhibit much greater resistance and heterogeneity compared to controlled experimental conditions [57]. Furthermore, the metabolic stability and effective delivery of these peptides present major obstacles. Proteases and peptidases in the body rapidly degrade AMPs, which reduce their therapeutic efficacy [58].

Peptidases display specific expression and activity patterns in each tissue, causing peptide degradation rates to vary depending on their location in the body. This specific degradation complicates the use of peptide drugs because peptidase activity in different tissues can drastically reduce their effectiveness and bioavailability [59,60]. Additionally, the potential toxicity and immunogenicity of these peptides require thorough evaluation to ensure patient safety [60]. In aqueous solutions, peptides are also vulnerable to degradation through oxidation, deamidation, and hydrolysis, which compromises their biological activity and effectiveness [61]. Various strategies have been explored to improve AMP stability and delivery. One approach is the design of peptide analogs with modified amino acid sequences to resist enzymatic degradation. Other strategies include incorporating D-amino acids, peptide cyclization, and developing peptidomimetics, and these compounds mimic peptide structures but offer superior stability [58,62].

In this context, Cong et al*.* [63] investigated the effectiveness of the peptide-mimetic polymer Gly-POX20 as an antimicrobial and antibiofilm agent against multidrug-resistant Gram-positive bacteria. This compound demonstrated potent antibiofilm activity and antimicrobial properties. In vitro studies showed that Gly-POX20 inhibited biofilm formation at concentrations 2 times the minimal inhibitory concentration (MIC), while eradicating mature biofilms required a concentration of 16 times the MIC. In comparison, traditional antibiotics like vancomycin failed to eliminate mature biofilms even at concentrations exceeding 1,000 times the MIC. In in vivo tests, Gly-POX20 was evaluated in a mouse keratitis model associated with MRSA biofilms on contact lenses. Results indicated that mice treated with Gly-POX20 experienced a significant reduction in ocular bacterial load, achieving reductions of up to 4.2 log colony-forming units (CFU) per eye compared to 6.3 log CFU observed with vancomycin. Additionally, treatment with Gly-POX20 resulted in significantly lower keratitis clinical scores (5.1) compared to vancomycin treatment (8.9), indicating greater therapeutic efficacy and reduced ocular inflammation. These results reflect superior therapeutic efficacy and reduced ocular inflammation compared to vancomycin. However, long-term toxicity, the potential development of resistance, and efficacy against mixed biofilms or in other types of ocular tissues remain unknown.

**Fig. 3.**
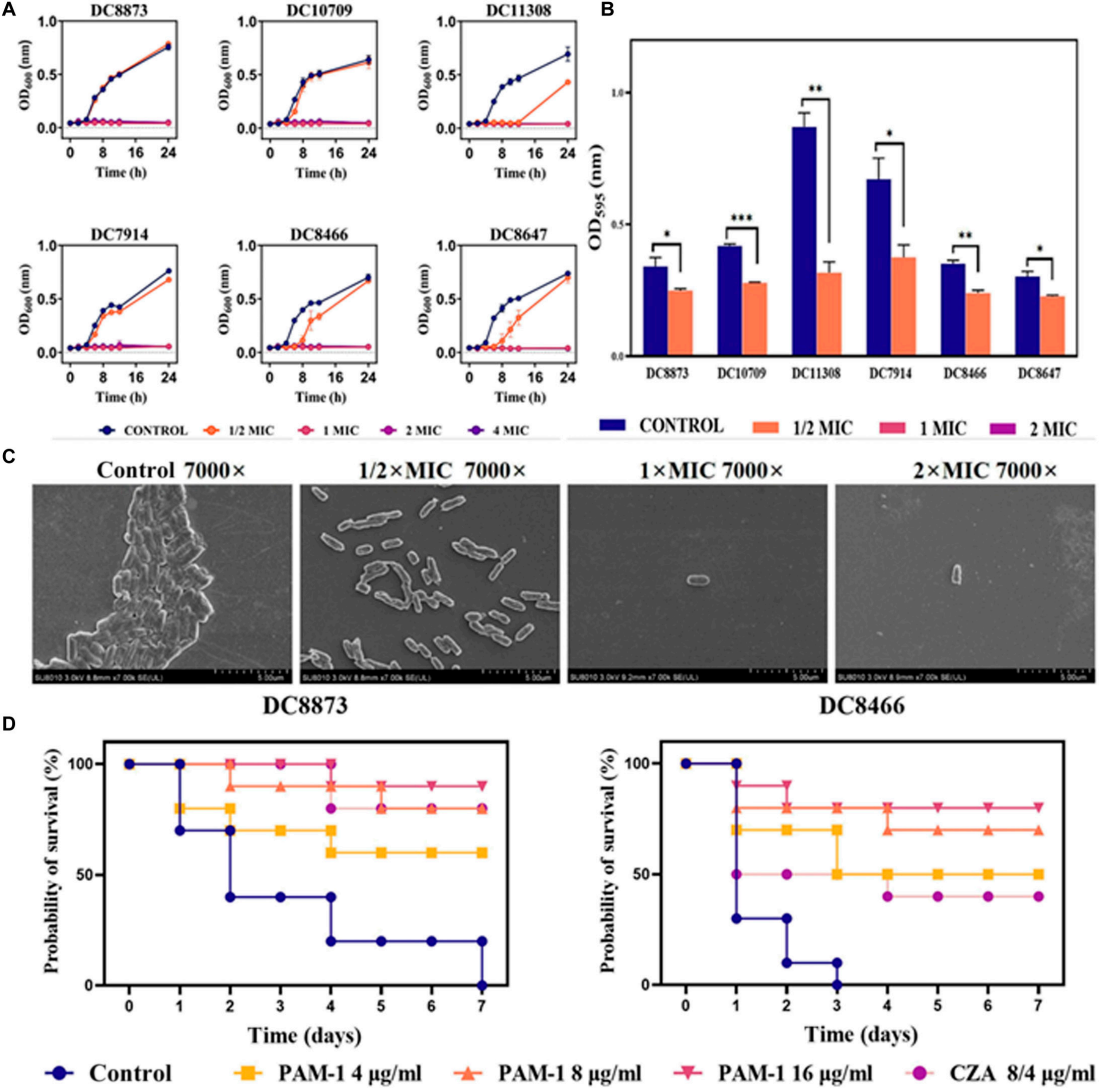
(A) Growth curves of PAM-1 treatment against 3 CZA-susceptible and 3 CZA-resistant *E. coli* strains. (B) Effect of PAM-1 on biofilm formation. SEM images of *E. coli* DC 8466 biofilm and bacterial morphology. (C) Results: Control, ^1^/_2_ MIC, 1 MIC, and 2 MIC, all observed at ×7,000 magnification. (D) Survival rate of *G. mellonella* for different therapies. Reprint published from an open access article by MDPI.

On the other hand, Han et al*.* [64] examined the effect of the AMP PAM-1 on *Escherichia coli* strains resistant to ceftazidime–avibactam (CZA). In vitro experiments revealed MICs between 2 and 8 μg/ml and a prolonged antibacterial effect for 12 h even at ^1^/_2_ MIC on the DC 11308 strain (Fig. 3A). Additionally, PAM-1 inhibited biofilm formation in various strains starting from ^1^/_2_ MIC (Fig. 3B). Scanning electron microscopy (SEM) analyses revealed that PAM-1 caused structural disruptions in biofilms, demonstrating its capability to destroy the bacterial matrix (Fig. 3C). In a *Galleria mellonella* infection model, PAM-1 increased the survival rate of larvae infected with CZA-resistant *E. coli* by over 50% after 7 d of treatment, surpassing the effect of CZA treatment alone (Fig. 3D). Furthermore, PAM-1 reduced the expression of inflammatory cytokines such as interleukin-1β (IL-1β) and tumor necrosis factor-α (TNF-α), demonstrating anti-inflammatory properties that could mitigate adverse responses during infections. Additional studies evaluated the stability of PAM-1 in the presence of serum, where it retained its efficacy, although its activity was significantly reduced in saline environments. These findings highlight the potential of PAM-1 as an alternative treatment for infections caused by antibiotic-resistant *E. coli*. This study highlights the effectiveness of PAM-1 in an invertebrate model, but extrapolation to mammals requires a more detailed investigation of its pharmacokinetics, toxicity, and immune system interactions, given the different physiological responses between insects and vertebrates.

Zhou et al*.* [65] investigated the effect of the AMP A20L against carbapenem-resistant *Klebsiella pneumoniae* (CRKP) in in vitro and in vivo models. A20L displayed a MIC of 4 to 8 μg/ml and a minimum bactericidal concentration ranging from 4 to >16 μg/ml. In biofilm studies, A20L inhibited formation in more than half of the strains at ^1^/_2_ MIC and partially eliminated mature biofilms in 2 of the 8 tested strains. In a *G. mellonella* infection model, A20L increased the survival rate of larvae infected with CRKP strains compared to the control group, indicating in vivo efficacy. Additionally, A20L was stable at different temperatures and in the presence of low serum concentrations, although its activity decreased in the presence of Ca^2+^, Mg^2+^, and high serum concentrations. Other relevant findings included the ability of A20L to increase bacterial membrane permeability, observed through electron microscopy and permeability assays, suggesting that the peptide acts by damaging the bacterial cell membrane. The research concludes that A20L shows potential for clinical applications in resistant infections, although factors like serum stability require improvements for future development. Although the observed efficacy is encouraging, the decreased activity in the presence of ions and serum underscores the need to optimize the formulation or structure of the peptide for clinical use, especially in environments with high levels of salts and proteins.

**Fig. 4.**
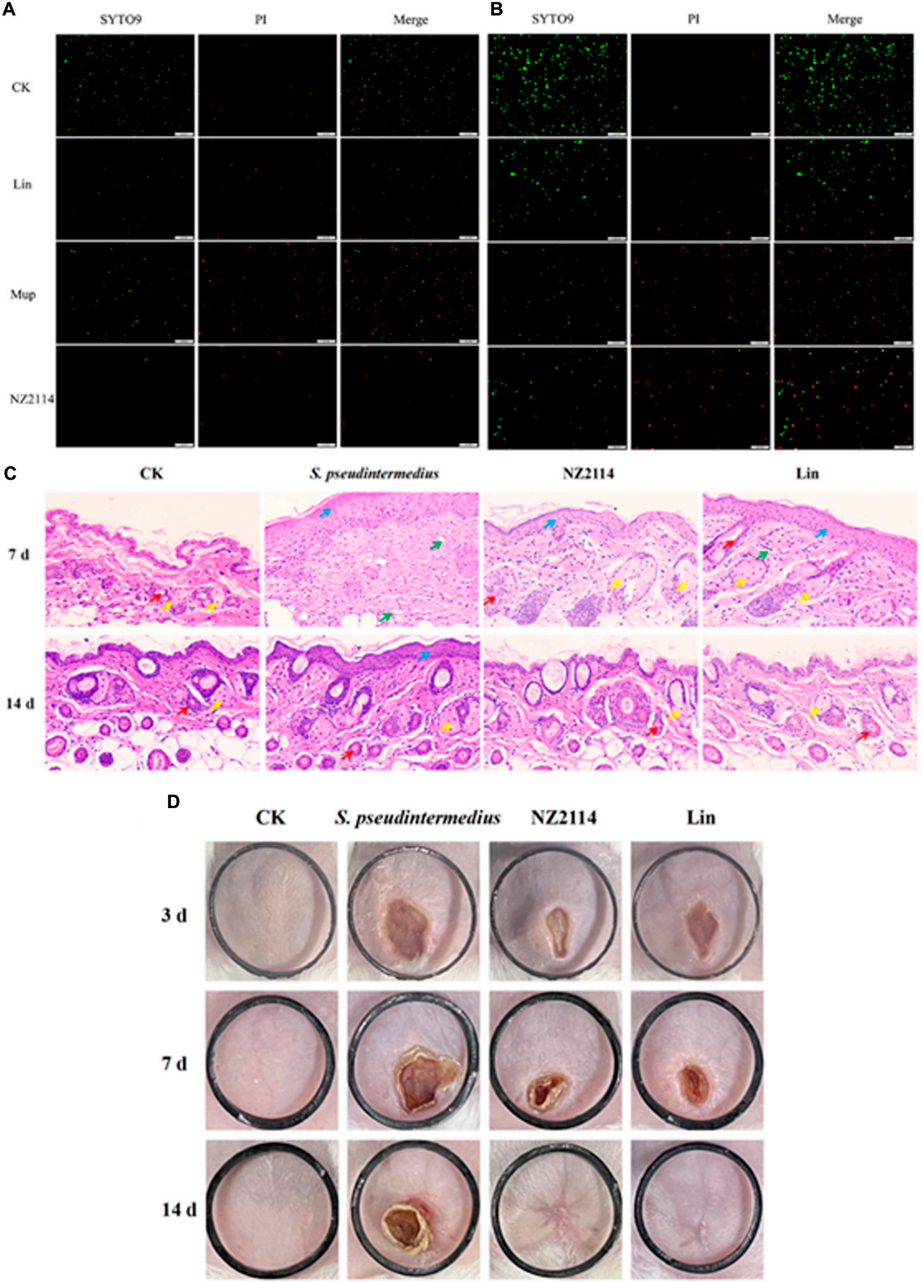
Observation of bactericidal effect by fluorescence microscopy. (A) *S. pseudintermedius* CGMCC 1.90024. (B) *S. pseudintermedius* CGMCC 1.90005. (C) Histological assays of mouse skin tissue at 7 and 14 d. CK, uninfected mice; *S. pseudintermedius*, infected mice without treatment; NZ2114, mice treated with NZ2114; Lin, mice treated with lincomycin. (D) Photographs of abdominal wounds on days 3, 7, and 14 after challenge with *S. pseudintermedius* CGMCC 1.90024. Reprint published from Zhang et al*.* [66], an open access article by MDPI.

Likewise, Zhang et al*.* [66] evaluated the efficacy of the AMP NZ2114 against *Staphylococcus pseudintermedius*, a common opportunistic pathogen in canines that can also affect humans. In vitro analysis showed a MIC of 0.23 μM, superior to mupirocin (MIC = 0.25 to 0.5 μM) and lincomycin (MIC = 4.34 to 69.41 μM). Fluorescence microscopy revealed that NZ2114 treatment caused marked disruption of the cell membrane of *S. pseudintermedius*, allowing propidium dye entry and indicating cell membrane destruction (Fig. 4A and B). Additionally, electron microscopy analysis demonstrated that NZ2114 caused damage to the cell membrane of *S. pseudintermedius*, resulting in cellular content loss and cell death. In biofilm assays, NZ2114 inhibited initial biofilm formation by 79.7% to 90.9% at a concentration of 16× MIC. In in vivo studies using a murine pyoderma model, NZ2114 significantly reduced bacterial load on the skin and decreased abscess area, accelerating tissue recovery. By day 14, the skin of mice treated with NZ2114 showed a 100% reduction in infection, whereas untreated mice still presented severe inflammation and abscesses (Fig. 4B and C). This study exemplifies the potential of NZ2114 for treating skin infections and reducing associated inflammation. Nevertheless, to extend its use to systemic infections, it will be necessary to investigate its stability, biodistribution, and possible immunomodulatory effects in a more complex physiological setting.

Despite these advancements, the administration of AMPs often requires invasive methods like intravenous injections due to their low oral bioavailability [61]. Overcoming these hurdles necessitates innovative formulation strategies and delivery systems that can maintain the activity of antibiofilm peptides within the human body while minimizing adverse effects [67]. Recently, advanced drug delivery systems have been developed to protect peptides from the harsh in vivo environment. These include encapsulation within biodegradable polymers, liposomes, and nanoparticles, which can shield the peptide from enzymatic degradation and provide controlled release [68,69]. However, each of these strategies must be optimized to balance peptide stability, targeted release, and minimization of potential toxicity or immunogenicity. Further research is essential to bridge the gap between promising laboratory findings and the successful clinical application of AMPs in combating antibiotic-resistant infections.

## Nanoparticles for AMP Delivery

**Fig. 5.**
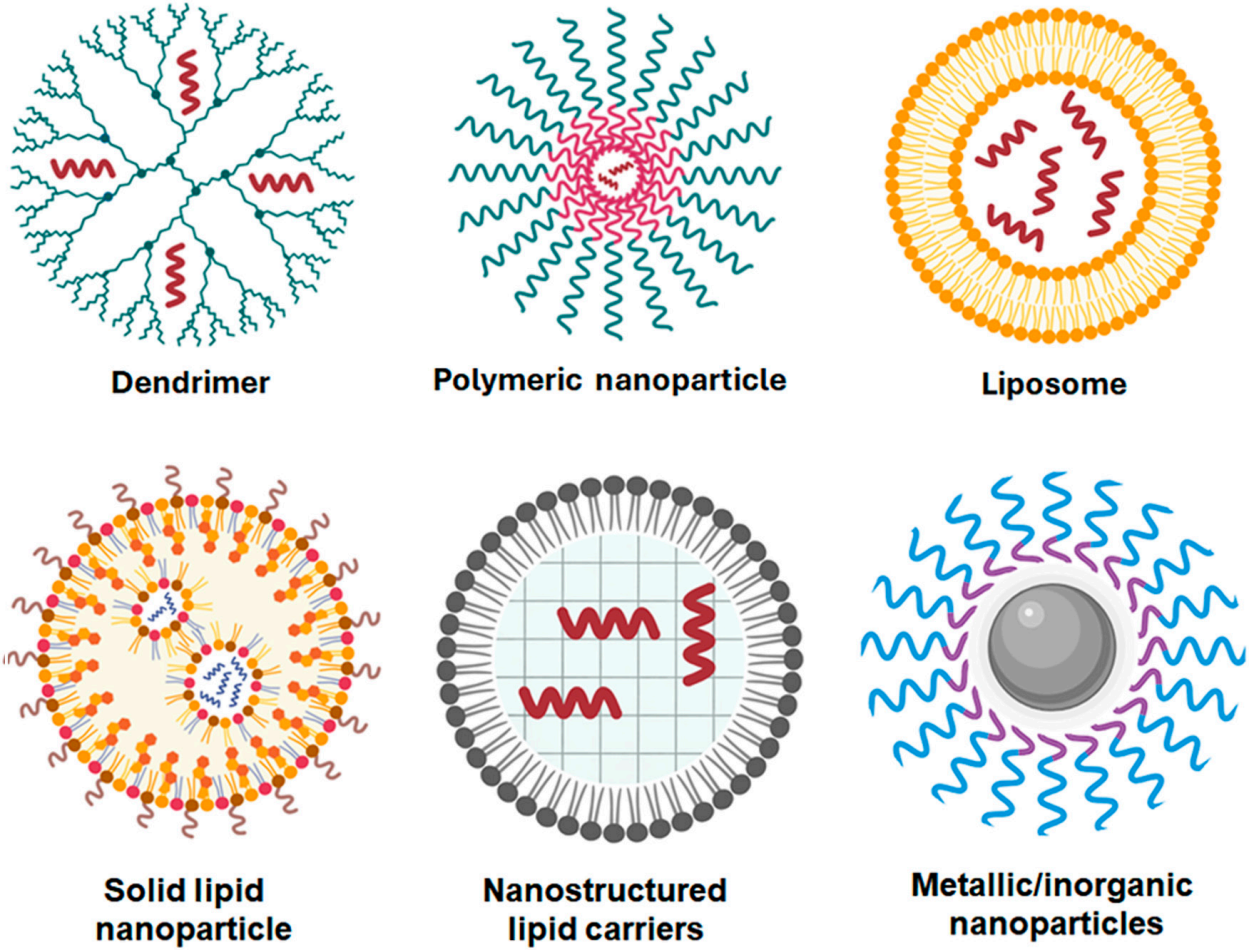
Types of nanoparticle delivery systems for antimicrobial peptides and biofilm inhibition. Designed BioRender.

Research on nanoscale carriers for delivering AMPs encompasses several categories: inorganic or metallic nanoparticles (such as gold, silver, and silica nanoparticles), polymeric nanoparticles (PNs) [including chitosan, hyaluronic acid, and poly(lactic-co-glycolic acid) (PLGA) nanoparticles], lipid-based nanoparticles [such as liposomes, solid lipid nanoparticles (SLNs), and nanostructured lipid carriers (NLCs)] [70] (Fig. 5), and other nanostructures like dendrimers, carbon nanodots, and quantum dots, mentioned in Table 2 [71].

**Table 2.** Types of nanoparticle delivery systems for AMPs and biofilm inhibition and its advantages and limitations

Nanoparticle	Composition	Properties	Advantages	Limitations	Ref.
Inorganic/metallic nanoparticles	Metal oxides	Range from 10 to 100 nm and can be composed of pure metals, metal salts, or metal oxides	When positively charged, they can establish strong bonds with bacterial envelopes, leading to cell rupture and leakage of cytoplasmic material, in addition to the penetration of metal ions into bacteria, which can cause metabolic disturbances. These nanoparticles exhibit anti-biofilm action by penetrating bacterial cell walls, disrupting quorum sensing, and interfering with the extracellular polymeric matrix.	Their use is limited by the toxicity of the reagents used in nanoparticle synthesis and their nonbiodegradability. Another obstacle to their application is the tendency of these particles to aggregate when dispersed, leading to a reduction in their antimicrobial activity.	[71,75]
Polymeric nanoparticles	Polymeric materials in form of nanospheres or nanocapsules	Range in size from 1 to 1,000 nm	It allows the controlled delivery of various types of drugs based on the variability of their matrix composition. The surface composition of polymeric nanoparticles (which can be functionalized with ligands) allows the administration of the drug in a specific location, minimizing side effects and increasing the efficacy of the treatment. In addition, they can be administered by a wide range of routes.	Most techniques using preformed polymers require organic solvents, which may pose toxicity risks and environmental concerns, necessitating solvent removal steps.	[73,76]
Dendrimers	Composed of 3 structural components: a core, monomeric branches, and terminal functional groups. The branches are linked to the core in a globular structure, and the terminal groups are external ligands attached to the branches.	Hyperbranched polymeric structures with a 3-dimensional architecture, typically ranging from 2 to 5 nm in size	The terminal groups can be composed of targeting structures, increasing the number of particles at the desired site of action. Their globular structure provides nanodomains with different properties between the core and the periphery, allowing drug encapsulation.	Low absorption by cancer cells and rapid clearance by the immune system	[78,79]
Liposomes	Spherical vesicular nanoparticles composed of phospholipids and cholesterol, forming a lipid bilayer around an aqueous core	Liposomal vesicles range in size from 0.025 to 2.5 μm and are classified according to the number of bilayers (lamellae) surrounding the aqueous core.	Liposomes offer several advantages, including their amphiphilicity, high solubilization capacity, good colloidal stability, protection of drugs from environmental factors, biodegradability, biocompatibility, and the ability to achieve intracellular distribution of substances. Additionally, their phospholipid composition gives them high affinity for biological membranes, significantly enhancing cellular drug uptake.	Susceptibility to chemical degradation, oxidation, and hydrolysis of the lipid bilayers, which can compromise the stability and quality of the system. Liposomes also face physical instability, including aggregation, fusion, or coalescence, which can alter vesicle size and lead to drug leakage. Other reported drawbacks include potential allergic reactions, high production costs, and long preparation times.	[80–82]
Solid lipid nanoparticles and nanostructured lipid carriers	Lipid-based nanoparticles that melt at temperatures above 40 °C	Solid lipid nanoparticles consist of a solid lipid matrix, while nanostructures lipid carriers are composed of solid lipids immersed in oil droplets, both stabilized by appropriate surfactants and capable of carrying hydrophilic or hydrophobic molecules.	They are biodegradable, nontoxic nanoparticles that can carry chemically diverse bioactives and can be surface-modified for targeted delivery. Their colloidal size and controlled release properties make them suitable for oral, ocular, and parenteral administration, with topical administration being particularly successful.	A common drawback of both nanosystems is their limited retention of polar peptides, due to their lipophilic composition.	[75,84,85]

### Inorganic/metallic nanoparticles

Nanoparticle systems can be classified according to their composition material and matrix properties into organic and inorganic systems. Inorganic nanostructures are typically composed of metal oxides obtained through the chemical reduction of metallic salts [72]. Metallic nanoparticles range from 10 to 100 nm and can be composed of pure metals, metal salts, or metal oxides. These nanoparticles exhibit a high surface-to-volume ratio, along with excellent optical and electric field properties, making them useful in bioimaging and sensor applications [73].

Due to electrostatic interactions, negatively charged bacterial cell walls attract positively charged nanoparticles. Both Gram-positive and Gram-negative bacteria have negatively charged cell walls, but the composition of their envelopes differs. Gram-positive bacteria possess a thick peptidoglycan layer, which is a cohesive mesh of *N-*acetylglucosamine and *N*-acetylmuramic acid residues, along with negatively charged teichoic acids. Gram-negative bacteria, on the other hand, have a thinner peptidoglycan layer and a phospholipid outer membrane containing lipopolysaccharides (LPSs), which further increase the negative surface charge of their cell envelopes. Positively charged metal-based nanoparticles establish strong bonds with bacterial envelopes, leading to cell rupture and increased permeability of cellular components. Additionally, metallic nanoparticles can release metal ions into the extracellular space, which, once inside the bacterial cell, can disrupt biological processes, induce reactive oxygen species (ROS) production, and bind to cellular structures such as proteins, DNA, and membranes. The interaction of metal ions with biological molecules is often nonspecific, involving coordination with nitrogen, oxygen, and sulfur atoms, thereby broadening the antimicrobial spectrum of this type of nanoparticle [74].

Regarding biofilms, metallic nanoparticles such as silver, gold, zinc, copper, and iron exhibit anti-biofilm effects by penetrating the bacterial cell wall, disrupting quorum sensing, and interfering with the extracellular polymeric matrix. These actions occur in conjunction with oxidative stress damage caused by these particles [73]. When associated with other substances, such as AMPs, the AMPs can be conjugated onto the surface of these nanoparticles, allowing for direct interaction with bacterial membranes. The antimicrobial efficacy of these nanomaterials is determined by their size, shape, and surface properties (such as zeta potential). However, their use is limited by the toxicity of the reagents used in nanoparticle synthesis and their nonbiodegradability. Another obstacle to their application is the tendency of these particles to aggregate when dispersed, leading to a reduction in their antimicrobial activity. This phenomenon can be mitigated by using water-soluble surfactants and polymers (e.g., chitosan, polyvinyl sulfate, or polysaccharides) to stabilize the nanoparticle system. An example of this strategy is silver nanoparticles stabilized by polyvinylpyrrolidone (a polymer) and surfactants like Tween 80 and SDS [71,75].

### Polymeric nanoparticles

PNs range in size from 1 to 1,000 nm and are composed of polymeric materials. They can exhibit 2 types of morphological structures: nanospheres and nanocapsules. Nanospheres consist of a continuous polymer network in which drugs can be adsorbed onto the surface or retained within the core. Nanocapsules, on the other hand, have a polymeric shell surrounding an oily inner core, where drugs can be either adsorbed on the surface or trapped within the core. The antimicrobial activity of PNs is achieved by inhibiting bacterial growth through interference with membrane biosynthesis and preventing biofilm formation and dispersal [73,76,77].

The use of PNs as drug carriers presents several advantages, including the ability to modify their composition, which enables controlled drug release based on the degradation rate of the polymer matrix, thereby improving bioavailability. The surface composition of PNs (which can be functionalized with ligands) allows for site-specific drug delivery, minimizing side effects from drug accumulation in nontarget tissues and increasing treatment efficacy. Moreover, PNs can be administered via various routes, including oral, nasal, topical, intraocular, and intravenous administration due to their small size [76,77].

The choice of polymer and synthesis method depends on the drug properties and the intended route of administration, as these factors influence the shape, size, and surface characteristics of the nanosystem, ultimately affecting drug activity. There are 2 main strategies for PN synthesis: preformed polymer dispersion and monomer polymerization. Most techniques using preformed polymers require organic solvents, which may pose toxicity risks and environmental concerns, necessitating solvent removal steps. Monomer polymerization, on the other hand, involves emulsifying hydrophobic monomers and inducing polymerization through initiator molecules, resulting in aqueous colloidal suspensions. Examples of PNs studied for antimicrobial activity include those made from polyethylene glycol (PEG), chitosan, and PLGA [73,76,77].

### Dendrimers

Dendrimers are hyperbranched polymeric structures with a 3-dimensional architecture, typically ranging from 2 to 5 nm in size. They are composed of 3 structural components: a core, monomeric branches, and terminal functional groups. The branches are linked to the core in a globular structure, and the terminal groups are external ligands attached to the branches. Each branching section is referred to as a generation, numbered sequentially from the core to the terminal group (e.g., G1, G2, and G3). Higher generations have more branches and a greater number of functional groups at the terminals, which are available for interactions with other molecules. Terminal groups can be composed of targeting structures, aimed at increasing the number of particles at the desired action site. Additionally, the terminals allow for multiple copies of a drug (or ligand) to be conjugated to the dendrimer periphery, thus exposing a large amount of the drug on the 3-dimensional surface, increasing interactions between the drug and bacterial cells and their receptors. The globular structure of dendrimers also provides nanodomains with different properties between the core and periphery, enabling drug encapsulation [78].

Dendrimers can overcome some limitations of traditional pharmaceutical formulations, such as rapid cellular absorption, targeted delivery, higher drug loading capacity, resistance to hydrolysis, and easier passage through biological barriers via transcytosis. These features make them promising candidates for overcoming the pharmacological limitations of AMPs. Cationic and amphiphilic dendrimers exhibit antimicrobial properties through electrostatic interactions between their positive charges and the negatively charged bacterial surface, leading to cell wall disruption. However, the main disadvantages of dendrimers include low absorption by cancer cells and rapid clearance by the immune system. The most studied dendrimers in medical research are poly(amidoamine) (PAMAM), poly(propylenimine), poly(l-lysine) (PLL), carbosilane, poly(phosphor-hydrazone) (PPH), and polyester dendrimers [78,79].

### Liposomes

Liposomes are spherical vesicular nanoparticles composed of phospholipids and cholesterol, forming a lipid bilayer around an aqueous core. Phospholipids are amphiphilic molecules composed of glycerol linked to a phosphate group (polar region) and 2 saturated or unsaturated fatty acid chains (nonpolar region). Liposomal vesicles range in size from 0.025 to 2.5 μm and are classified according to the number of bilayers (lamellae) surrounding the aqueous core. They can be unilamellar (with a single phospholipid bilayer) or multilamellar (with multiple bilayers separated by water). The number of lamellae and the size of the liposomes directly affect the drug half-life. Due to their amphiphilic nature, liposomes can encapsulate both hydrophilic substances (in their aqueous core) and hydrophobic substances (within the lamellae through van der Waals interactions). The properties of liposomes vary depending on their composition (types of phospholipids and glycerol content), number of lamellae, size, surface charge, and preparation method [80–82].

Liposomes offer several advantages, including their amphiphilicity, high solubilization capacity, good colloidal stability, protection of drugs from environmental factors, biodegradability, biocompatibility, and the ability to achieve intracellular distribution of substances. Additionally, their phospholipid composition gives them high affinity for biological membranes, significantly enhancing cellular drug uptake [80,81,83]. Liposome synthesis can be achieved using various techniques, depending on the desired size, composition, lipid properties, and preparation scale. Generally, these techniques are divided into 2 categories: passive loading (where compounds are introduced before or during liposome production) and active loading (where compounds are introduced after vesicle formation) [81].

Despite their advantages, liposomal nanosystems have several limitations, such as susceptibility to chemical degradation, oxidation, and hydrolysis of the lipid bilayers, which can compromise the stability and quality of the system. Liposomes also face physical instability, including aggregation, fusion, or coalescence, which can alter vesicle size and lead to drug leakage. These factors shorten the half-life of liposomes, affecting treatment efficacy. Other reported drawbacks include potential allergic reactions, high production costs, and long preparation times [81,82].

### SLNs and NLCs

SLNs and NLCs are lipid-based nanoparticles that melt at temperatures above 40 °C (body temperature). SLNs consist of a solid lipid matrix, while NLCs are composed of solid lipids immersed in oil droplets, both stabilized by appropriate surfactants and capable of carrying hydrophilic or hydrophobic molecules [75,84]. SLNs and NLCs were developed as alternatives to liposomes due to their higher stability, better encapsulation efficiency, longer shelf life, and scalability for large-scale production [84]. Both SLNs and NLCs offer numerous advantages: They are biodegradable, nontoxic nanoparticles that can carry chemically diverse bioactives and can be surface-modified for targeted delivery [75]. Their colloidal size and controlled release properties make them suitable for oral, ocular, and parenteral administration, with topical administration being particularly successful, which is why these nanoparticles are widely used in commercial formulations for cosmetic applications [84,85].

SLNs can be produced by 2 main techniques: high-pressure homogenization and microemulsion-based methods. Both methods avoid the use of toxic organic solvents, which is a key advantage of SLNs. They are also highly stable, even in the gastrointestinal tract, and can accommodate high drug loads of lipophilic compounds. However, SLNs exhibit some disadvantages, such as low loading capacity for AMPs, a tendency toward gelation and polymorphic transformations, premature drug release during storage, and low encapsulation efficiency due to the solid lipid matrix’s crystalline structure. NLCs were developed as second-generation lipid carriers to address issues associated with SLNs, such as limited peptide loading capacity and drug expulsion during storage. NLCs provide higher encapsulation efficiency, more uniform drug release, and improved permeability and bioavailability [86]. A common drawback of both SLNs and NLCs is their limited retention of polar peptides, due to their lipophilic composition. Techniques such as double-emulsion methods and cold and hot high-pressure homogenization are employed to improve the transport efficiency of these molecules [84]. Some applications using nanotechnology and AMPs to inhibit biofilms with promising results are shown in Table 3.

**Table 3.** Examples of peptide-carrying nanosystems applied to microorganisms and biofilms. NR, not reported.

Nanosystems	AMP	Bacteria	Biofilm inhibition of the nanosystem	MIC	Mechanism of action	Ref.
Col-loaded poly(lactideco-glycolide) (PLGA) nanoparticles (NPs) Nano-embedded microparticles and Col- PVA NEM	Colistin	*P. aeruginosa*	50%	NR	It binds to lipopolysaccharide in the outer membrane of Gram-negative bacteria.	[101]
Hybrid poly(sulfobetaine) methacrylate/polymyxin B nanoparticles (pSBMA@PM NPs)	*Polymyxin B*	*P. aeruginosa*	80%	NR	Membrane disruption combined with “invisibility” to the bacterial defense system, which fails to recognize them as a threat.	[102]
Colistin-monoolein liquid crystal nanoparticles	Colistin	*P. aeruginosa*	At 60 μg/ml, *P. aeruginosa* biofilm was nearly eradicated.	0.5 μg/ml	This nanosystem was able to increase drug penetration through the biofilm matrix by forming a coating that protects colistin from electrostatic interactions.	[103]
Liposome-peptide	RRWRIVVIRVRRC	*P. aeruginosa*	50%	10.32 μg/ml		[104]
Mesoporous silica nanoparticles with large pores coated with melittin peptide and ofloxacin	Melittin peptide	*P. aeruginosa*	>88% after 10 min and >96% after 30 min of treatment		It was able to increase drug penetration through the biofilm matrix by destabilization of the structure, resulting in higher doses of drug being released inside the biofilm.	[105]
C7-3-peptide-loaded chitosan nanoparticles	C7-3 (H-ACNYCRLNLWGGGS-NH2)	*N. gonorrhoeae*	52%	0.19 mM/ml	The mechanism of action of chitosan has not yet been elucidated, but one hypothesis is that the positively charged chitosan interacts with the negative charge of the lipopolysaccharide of the bacterial cellular membrane, causing destabilization of the membrane and leakage of intracellular material.	[106]
	C7-3m1 (H-ACNYSRLNLWGGGS-NH2)		42%	0.26 mM/ml		
	C7-3m2 (H-ACSY CRLNLWGGGSNH2)		32%	0.099 mM/ml		
Poly-l-lysine functionalized graphene−silver nanocomposites	Poly-l-lysine	*S. aureus*	>80%	8 μg/ml	Oxidative stress through ROS production, inhibition of enzyme synthesis by targeting the respiratory chain enzyme dehydrogenase, alteration of the cell membrane potential, and release of intracellular components through pore formation in the cell wall.	[107]
Gold nanocomposite, functionalized with antimicrobial peptide, Pediocin AcH, and Listeria adhesion protein (LAP)	Pediocin AcH	*L. monocytogenes*	24–31%	NR		[108]
Polycationic peptide functionalized graphene–silver nanocomposite	ε-Poly-lysine	*P. aeruginosa*	95%	5 μg/ml	Oxidative stress through ROS production, inhibition of enzyme synthesis by targeting the respiratory chain enzyme dehydrogenase, alteration of the cell membrane potential, and release of intracellular components through pore formation in the cell wall.	[109]
		*E. coli*	93%			
Liquid crystalline systems for KSL-W peptide oral administration	Decapeptide KSL-W (NH3+ -Lys-Lys-Val-Val-Phe-Trp-Val-Lys-Phe-Lys-CONH2)	Multispecies oral biofilm	100%	NR	Not yet fully elucidated	[110]
Esc(1-21)-coated AuNPs	Esculentin-1a {a(1-21)NH2 [Esc(1-21)], GIFSKLAGKKIKNLLISGLKG-NH2}	*P. aeruginosa*	50%	0.08 μM	Primarily disrupting the anionic microbial membrane, leading to the leakage of cytosolic components.	[111]
BRBR chitosan-based nanoparticles	RBRBR	*S. aureus*	86.5–98%	5 mg/ml	The positive charge enhanced binding to microbial membranes, facilitating stronger electrostatic interactions between the nanosystem and its target cells.	[112]
Plectasin derivative NZ2114 delivery mediated by polylactic glycolic acid nanoparticles	Plectasin derivative NZ2114	*Staphylococcus epidermidis*	9–100%	4–8 μg/ml	The NZ2114-NP particles, with a size of 178 nm (less than 350 nm), can effectively diffuse through biofilm pores. Additionally, their positive charge enhances biofilm disruption, facilitating deeper penetration of the particles.	[113]
LL37-loaded chitosan nanoparticles	LL37	MRSA	-	-	It inhibited MRSA biofilm attachment and disrupted its maturation process, which was attributed to the down-regulation of the icaA gene.	[114]
Hyaluronic acid-based nanogel for NO and peptide delivery	RKKKKLLRKKC	*E. coli*	-	1.56 μg/ml	The results of the molecular dynamic simulation suggest that peptide as co-antibiofilm agent can serve as an inhibitor of *SarA*-mediated regulation, thus limiting biofilm formation. The excellent binding affinity and stability of the AMP to *SarA* might have contributed to a better antibiofilm efficacy of the NO co-loaded nanogel.	[115]
		MRSA	12.5-fold reduction in biofilms	0.78 μg/ml		
		*P. aeruginosa*	24-fold reduction in biofilms	0.39 μg/ml		
AMPs modified silver nanoparticles	Sequence not mentioned	*E. coli*	5.99%	25 μg/ml	The exploration of antibacterial mechanism indicated that the nanocomposite may preclude the formation of biofilm by inhibiting the transcription level of biofilm-related genes.	[116]
		MRSA	5.74%			
		*P. aeruginosa*	5.23%			
Gold nanoparticles tethering AMPs	Ura56	MRSA	90% inhibition of initial biofilms and 80% reduction of preformed biofilms	0.13−1.25 μM	Kill bacteria through a membrane-disruption mechanism and increased overall reactive oxygen species production	[117]
		*E. coli*				
		Multidrug-resistant *P. aeruginosa*				
		*Acinetobacter baumannii*				
Jellein-I-conjugated gold nanoparticles	Jellein-I-Cys (Pro-Phe-Lys-Ile-Ser-Ile-His-Leu-Cys-NH_2_)	MRSA *S. aureus*	72%	94 μM	Jellein-I-Cys-GNPs can act through multiple mechanisms, such as inhibiting protein and DNA synthesis and disrupting bacterial membranes, leading to the absence or slower development of resistance in bacteria	[118]
pHly-1 nanoparticles	pHly-1	*Streptococcus mutans*	-	5.5–11 μM	Peptide pHly-1 adopts random coil conformation under acidic conditions and forms nanoparticles. It kills bacteria mainly via cell membrane disruption mechanisms.	[119]
Immobilized arginine/tryptophan-rich cyclic dodecapeptide on reduced graphene oxide anchored with manganese dioxide	Cyclic dodecapeptide peptide (Cdp-1)	*P. aeruginosa*	99%	31.25 μg/ml	The cationic nature of AMP promotes electrostatic interaction with the negative surface of *P. aeruginosa* and improves binding to the phospholipid membrane, causing depolarization of the cell membrane, which can generate holes and cause leakage of the bacterial cell content after the internalization of the peptide.	[120]
	Cdp-2			62.5 μg/ml		
	Cdp-3			15.63 μg/ml		
7e-SMAMP liposomes	7e-SMAMP (synthetic mimic of antimicrobial peptide)	*P. aeruginosa*	86%	25.00 μg/ml	Its mechanism of action is related to the disruption of lipid packaging within the bacterial cell membrane.	[121]
		*S. aureus*	48–99%	0.78–1.56 μg/ml		
		MDR *S. aureus*		3.13–12.50 μg/ml)		
Liposome-encapsulated tobramycin and IDR-1018 peptide	IDR-1018 peptide (innate defense regulator peptide 1018)	*P. aeruginosa*	Biofilm formation was significantly reduced (*P* < 0.05) at concentrations of ≥4 and ≤32 μg/ml for *P. aeruginosa* and ≤32 μg/ml for MDR *P. aeruginosa* when loading tobramycin into liposomes.	≥256 μg/ml	The mechanism of IDR-1018 as an anti-biofilm is due to the binding and destruction of second messenger stress-induced nucleotides (p)ppGpp, which can promote the formation of biofilm.	[122]
		MDR *P. aeruginosa*				

### How about in vivo nanotechnology studies?

Yu et al*.* [87] developed a mesoporous silica nanoparticle (MSN) platform for the coadministration of the AMP melittin (MEL) and the antibiotic ofloxacin (OFL), demonstrating significant antibiofilm activity. This system uses a co-assembled host–guest particle structure, allowing the controlled release of both agents through heat and alternating magnetic field (AMF) stimuli. In in vitro experiments, the H-MEL + G-OFL nanoparticles reduced biofilm biomass by over 97%, significantly surpassing the 69% reduction achieved with free agents, and eliminating 100% of pathogenic cells compared to 78% achieved with conventional treatments. Electron microscopy analysis confirmed effective dispersion and destruction of biofilm cells in the presence of H-MEL + G-OFL, contrasting with the dense structure observed in untreated biofilms. In in vivo implantation models, implants treated with this system completely eradicated bacterial biofilm without triggering inflammation, evidenced by normal cytokine levels in treated tissues compared to elevated levels in the control group. These results suggest that the H-MEL + G-OFL platform is a promising option for the effective eradication of biofilms in in vivo applications, especially in implanted medical devices.

Mahmoud et al*.* [88] studied the efficacy of PLGA nanoparticles modified with the BAR peptide (BNPs) to inhibit oral biofilms in a murine periodontitis model. These nanoparticles are designed to disrupt the formation of biofilms of *Porphyromonas gingivalis* (Pg) and *Streptococcus gordonii* (Sg), key bacteria in the pathogenesis of periodontal diseases. Characterization of BNPs showed a diameter of 87.9 ± 29.4 nm in the nonhydrated state and 333.8 ± 17.8 nm in the hydrated state, reflecting an expansion upon water contact. Additionally, the zeta potential was −10.3 ± 0.9 mV compared to −22.6 ± 1.2 mV in unmodified nanoparticles, confirming effective conjugation with the BAR peptide and suggesting reduced particle aggregation. This optimized design enables BNPs to provide controlled release and enhanced adhesion in the oral environment. In in vivo tests, BNPs significantly reduced alveolar bone loss and IL-17 expression, a marker of inflammation, compared to free BAR use. At a concentration of 0.7 μM, BNPs reduced bone loss to nearly the level of uninfected animals, while free BAR was less effective at the same dose. In terms of safety, BNPs proved to be nontoxic at concentrations of 1.3 to 3.4 μM, showed no hemolytic activity in sheep erythrocytes, and maintained over 90% viability in gingival epithelial cells. Additionally, normal levels of apoptosis, adenosine triphosphate (ATP), and lactate dehydrogenase (LDH) were observed compared to untreated cells. These findings suggest that BNPs represent a promising biocompatible platform for oral biofilm treatment and periodontal inflammation management, with potential for clinical applications in oral infection prevention.

Zhang et al*.* [89] investigated the use of stimulus-sensitive antibacterial peptide nanoparticles to combat bacterial biofilm in dental caries, achieving significant results in in vivo models. The peptide pHly-1, derived from spider venom and modified to be dual-sensitive to pH changes and lipid binding, demonstrated advanced efficacy in inhibiting biofilms and preventing caries without affecting oral microbial diversity or damaging healthy tissues. These nanoparticles (pHly-1 NPs) showed pH-dependent antibacterial activity, adapting to the acidic environment characteristic of caries and adopting a helical conformation that disrupts bacterial membranes. In neutral conditions, pHly-1 forms nanofibers with low cytotoxicity, ensuring a safe profile for oral and gastric tissues. In nanoparticle characterization, dynamic light scattering analysis revealed that pHly-1 NPs formed particles of approximately 40 nm at pH 4.5, while their zeta potential (ζ) ranged from 17.9 ± 2.25 mV in acidic pH to 6.9 ± 0.18 mV in neutral pH, indicating a high positive charge in acidic environments, crucial for binding and disrupting *Streptococcus mutans* bacterial membranes. In vivo efficacy experiments showed that pHly-1 NPs not only reduced biofilm formation on dental surfaces in rat models but also effectively suppressed caries progression without adverse effects on body weight or on oral and gastric tissues of the subjects. Given the limited number of in vivo studies exploring the combination of nanotechnology and AMPs against biofilm-forming bacteria, it is imperative to act swiftly and advance research in this area before it becomes too late to effectively address the escalating threat of biofilm-related infections and antimicrobial resistance.

## Challenges and Future Directions

AMPs hold great therapeutic potential; however, their widespread application is hindered by significant challenges, including stability issues and poor compatibility with biological barriers [14,54]. AMPs are highly susceptible to enzymatic degradation and often exhibit short half-lives in systemic circulation, limiting their effectiveness in clinical applications. Additionally, their inability to efficiently penetrate mucosal layers, biofilms, and cellular membranes reduces their bioavailability. These limitations necessitate innovative solutions to enhance AMP performance in biological systems.

Chemical modifications have emerged as a key strategy to improve AMP stability and efficacy. Cyclization of peptides and incorporation of nonproteinogenic amino acids have shown promise in enhancing enzymatic resistance and extending peptide half-life [90]. Similarly, the immobilization of AMPs onto nanoparticles has demonstrated enhanced stability and antimicrobial activity while providing targeted delivery. Advances in engineering nanoparticle size, shape, and surface properties have addressed toxicity concerns, such as oxidative stress and immune activation, improving their safety profiles [68,69,90]. Metallic nanoparticles, such as gold, have shown great potential; however, Rajchakit and Sarojini [90] highlighted the importance of synthesizing well-defined shapes and sizes to minimize toxicity risks.

Recent innovations, such as hydrogel-based AMP delivery systems, offer promising alternatives to address nanoparticle-related limitations. Hydrogels, characterized by their high water content, biocompatibility, and biodegradability, allow for controlled drug release while improving AMP stability and reducing side effects Ahmad et al. [91]. The hydrogel-conjugated systems enhance biodisponibility and provide an adaptable platform for delivering AMPs in diverse therapeutic contexts. This technology presents a highly versatile solution that could overcome many existing challenges associated with AMP and nanoparticle systems.

Looking ahead, the future of AMPs lies in interdisciplinary research that integrates advanced technologies. Computational modeling and artificial intelligence (AI)-driven design could optimize AMP–nanoparticle conjugates, while CRISPR-based systems may enhance AMP specificity and reduce off-target effects. Additionally, refining production methods to lower costs and improve scalability will be critical for the clinical translation of AMP-based therapies. Despite the challenges, the rapid advancements in chemical modifications, nanoparticle delivery systems, and hydrogel technologies provide a clear path forward for the broader application of AMPs in combating multidrug-resistant infections.

## Conclusions

Innovative therapeutic strategies are essential to combat biofilm-forming bacterial infections, which are highly resistant to current treatments. AMPs show great potential for destabilizing or inhibiting biofilms, but their instability, short half-life, and challenges in administration limit their clinical use. Nanotechnology offers a promising solution by enhancing the stability, absorption, and antimicrobial efficacy of AMPs, with metallic and polymeric nanoparticles providing biofilm disruption, controlled release, and targeted delivery. Despite these advancements, challenges like nanoparticle toxicity, stability, and encapsulation difficulties remain. Integrating hydrogels into nanoparticle systems improves biocompatibility and controlled release, representing a viable strategy to address these issues. Future efforts should focus on optimizing and personalizing these technologies to ensure efficacy. In conclusion, combining nanotechnology and AMPs expands treatment options for biofilm-related infections, paving the way for more precise and effective therapies, although continued research is needed to overcome existing limitations.
